# A systematic review of anti-cancer roles and mechanisms of kaempferol as a natural compound

**DOI:** 10.1186/s12935-022-02673-0

**Published:** 2022-08-20

**Authors:** Elham Amjad, Babak Sokouti, Solmaz Asnaashari

**Affiliations:** Biotechnology Research Center, Tabriz University of Medical Sciences, Tabriz, Iran

**Keywords:** Anti-cancer, Natural compounds, Kaempferol, Mechanisms, Signaling pathways

## Abstract

It has been shown in multiple experimental and biological investigations that kaempferol, an edible flavonoid generated from plants, may be used as an anti-cancer drug and has been shown to have anti-cancer properties. Many signaling pathways are altered in cancer cells, resulting in cell growth inhibition and death in various tumor types. Cancer is a multifaceted illness coordinated by multiple external and internal mechanisms. Natural extracts with the fewest side effects have piqued the attention of researchers in recent years, attempting to create cancer medicines based on them. An extensive array of natural product-derived anti-cancer agents have been examined to find a successful method. Numerous fruits and vegetables have high levels of naturally occurring flavonoid kaempferol, and its pharmacological and biological effects have been studied extensively. Certain forms of cancer are sensitive to kaempferol-mediated anti-cancer activity, although complete research is needed. We have endeavored to concentrate our review on controlling carcinogenic pathways by kaempferol in different malignancies. Aside from its extraordinary ability to modify cell processes, we have also discussed how kaempferol has the potential to be an effective therapy for numerous tumors.

## Introduction

Cancer is an irregularity in the proliferation of various cells in different body tissues and is referred to as a group of diseases with an unmanageable growth and abnormal mechanism of cell division [1]. At the same time, there is always a balance between cell division, death, and differentiation [2]. The exact reasons for cancer are still unknown. Still, genetic and environmental factors such as ionizing radiations, viral infections, chemical and toxic substances, or excessive sunlight that disrupt cell function can cause abnormalities in the cell nucleus [3, 4]. The body's defense immune response is insufficient to manage the condition [2].

Biology, environment, lifestyle, and healthcare organization are central to preventing cancer and other chronic diseases. However, all of these issues are limited by environmental factors such as economic context, agricultural dimensions, nutrition, pollution, social condition, and education of individuals that can make healthy behavioral choices impossible [5–7].

More than 200 types of cancer are known. Many factors can lead to abnormal cell growth and eventually cancer, but what is important is early detection of cancer, which raises hopes for the effectiveness of the treatment [8–12]. It is not uncommon to utilize a mix of the most prevalent cancer therapies (such as surgery or chemotherapy) to treat the disease, depending on the patient's natural state and the kind of cancer [13–15]. Due to the adverse effects and drug resistances of mentioned treatments, natural compounds have been considered in the prevention and treatment of cancers and also reducing the chemo-radiotherapies side effects [15–17]. The use of natural compounds in treating various diseases has been prevalent since ancient times. About 80% of people worldwide use these compounds and are widely dependent on them [18]. These structures target the tumor cells by regulating cell death pathways, including apoptosis and autophagy [19]**.**

**Fig. 1:**
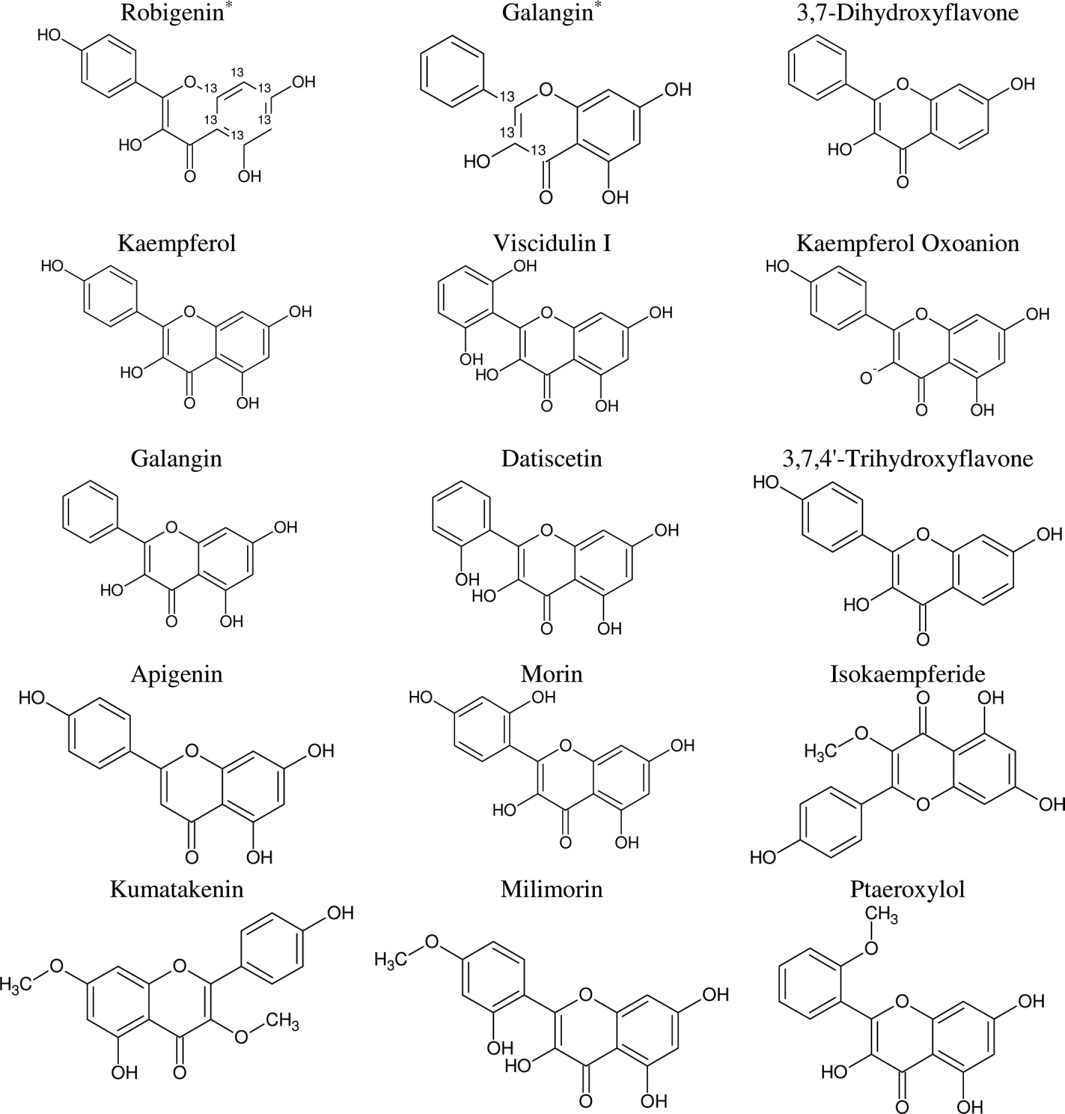
Kaempferol's chemical structure and its analogous compounds using the Tanimoto similarity index of 97% in PubChem database (^*^[13C6]-Robigenin and Galangin-13C3 have Carbon 13 (.^13^C))

One of the most common flavonoid compounds in plants is a tetrahydroxyflavone from the flavonol family known as kaempferol (i.e., 3,4′,5,7-tetrahydroxyflavone), as shown in Fig. 1, along with its potential analogous structures. It is found in many plants, particularly edible or medicinally important [20]. Previous epidemiological research shows kaempferol has been linked to various cardiovascular, cancer, inflammatory, neurodegenerative, and obese diseases [21]. Researchers have well-described kaempferol's chemopreventive and therapeutic activities in previous studies [15, 22]. In this research, we will conduct a comprehensive investigation and discussion to determine how kaempferol may be used to prevent and treat different cancer types.

## Pharmacokinetics of kaempferol

The biological and pharmacological effects of flavonoid structures are related to their original characteristic mechanisms of action and the activity of their metabolites due to their widespread and rapid biotransformation [23, 24]. Due to the variety of the binding substituents, most natural flavonoid structures, such as kaempferol, are commonly presented and consumed as glycoside forms [20, 25]. Although the high polarity of the attached glycosyl groups to the aglycon reduces their absorption, previous studies have revealed that flavonoid glycosides absorption without hydrolysis is feasible [26]. The lipophilic nature of this structure leads to passive and facilitated diffusion absorption; however, scientific research demonstrated that it might also be absorbed as an active transport process [27, 28]. Conjugation enzymes metabolize kaempferol to glucuronides and sulfoconjugates in the small intestine. It can also be hydrolyzed by colon microflora and converted to simple phenolics (e.g., 4-hydroxyphenyl acetic acid, phloroglucinol, and 4-methyl phenol) to absorb or exert in feces.

Moreover, the absorbed part of kaempferol is metabolized into glucuronides and sulfo-conjugates in the liver. Finally, the conjugated forms of kaempferol create phenolic chemicals in the colon, and kaempferol and its glycoside derivatives are eliminated in the urine [20, 28]. Earlier studies demonstrated that 3- glucuronide conjugate of kaempferol was the main detected component in plasma and urine. Likewise, the urine excretion of consumed kaemferol was approximately 1.9% to 2.5%. [29, 30]. Following oral ingestion of kaempferol, many human investigations have shown that the concentration of the compound in plasma is confined to nanomolar levels. For example, in a 2002 study by Radtke, the plasma concentration of kaempferol was reported to be 10.7 nM, while the intake amount was 4.7 mg/day [31]. Another research found that ingesting 27 mg of kaempferol in the form of daily tea resulted in a plasma concentration of 52 nM (15 g/L), which was attained by consuming 52 nM (15 g/L) of kaempferol [30]. A plasma concentration of 57.86 nM of kaempferol was measured in a dietary intervention study on 92 healthy students (mean age: 24.16 years; mean BMI: 21.31 kg/m2) [32].

## Anti-cancer activities of kaempferol in literature cancer types

A thorough review of the Scopus database was carried out on different types of cancer using “kaempferol” as their treatments, which will be described as follows.

### Bladder cancer

According to the GLOBOCAN latest report, bladder cancer considers 3% of diagnoses of global cancer and mainly occurs in developed countries [33].

Urinary tract carcinoma is the most frequent urinary system cancer [34]; Xie et al. demonstrated kaempferol's preventative and therapeutic benefits from human bladder cancer with EJ cells. They reported significant anti-proliferative and anti-migration activities of this natural flavonol. The apoptotic mechanism activated by kaempferol is connected to the PTEN tumor suppressor gene through the PI3K/Akt signaling pathway [35]. In the other study, the assessment of MTT assay on two different bladder cancer cell lines (5637 and T24 cells) demonstrated that kaempferol increased cell cytotoxicity. Still, the analysis of flow cytometry, DNA ladder, and TUNEL assays resulted in significant kaempferol-induced apoptosis and cell cycle arrest. In vivo investigations (subcutaneous xenografted mouse models) supported these in vitro findings, with kaempferol-treated animals showing less cancer formation, whereas apoptotic markers are elevated. The research revealed that kaempferol might suppress cell growth by significantly downregulating the c-Met/p38 signaling pathway [36]. Kaemferol altered DNA methylation by creating 103 gene-specific differential DNA methylation locations (dDMPs), as was reported in a cell line research (using 5637 and T24 cells) and a 31-day in vivo investigation in nude mice with bladder cancer distress. The study concluded that kaempferol could promote the DNMT3B degradation through the ubiquitin–proteasome pathway and introduced as a novel DNMT3B inhibitor [37]. Wu and colleagues discussed the radical scavenging activity of kaempferol, its function in stimulating antioxidant enzymes, controlling ROS formation, and the anti-hemolytic effects of lipid peroxidation. Their study showed that kaempferol was a potent inhibitor in bladder cancer with high safety on normal cells. Besides, it suppressed the phosphorylation of AKT, CyclinD1, CDK4, Bid, Mcl-1, and Bcl-xL, while boosting the expression of p-BRCA1, ATM, p53, p21, and p38, as well as Bax and Bid. It also promoted apoptosis and S phase arrest in EJ bladder cancer cells while decreasing proliferation [34].

### Bone cancer

Primary bone cancers are the most severe type of bone cancers, consisting of about 0.2% of all worldwide malignancies derived from primitive mesenchymal cells involving several subtypes of osteosarcoma chondrosarcoma Ewing sarcoma [38]. Previous research on osteosarcoma cells showed that kaempferol might increase the expression of Box protein and decrease Bcl-2 protein, decrease mitochondrial membrane potential and increase caspase-3, -7, and -9 activities in the U-2 OS cell line. A rise in AIF protein levels was also observed, indicating that apoptosis was induced through a caspase-independent mitochondrial mechanism. The endoplasmic reticulum stress pathways are the other mechanism induced by kaempferol in the human osteosarcoma cell line. In a study, the in vivo evaluation confirmed the antitumor effects of this bioflavonoid in mice models [39]. In another research, kaempferol inhibited cell metastasis in U-2 OS cells by inhibiting various signaling pathways (e.g., ERK, AP-1, JNK, and p38) [40]. When osteoblasts stopped producing osteoclastogenic cytokines and osteoclast precursor cells stop differentiating, kaempferol had an anti-osteoclastogenic impact on bone. It is due to antagonizing the TNF receptor family action on bone cells [41].

### Breast cancer

Female breast cancer accounts for 11.7% of all cases and 6.9% of cancer deaths globally [42]. There are several studies on kaempferol's effects and possible mechanisms on breast cancer. In 2004, Hung published an article that showed the significant efficacy of kaempferol on the impairment of the estrogen receptor-α and blocking the estradiol-induced cell proliferation, while estrogen receptor (ER)-negative breast cancer cells didn't show growth resistance toward the kaempferol [43]. In some in vitro and in vivo studies, kaempferol is a phytoestrogen that inhibits triclosan and exhibits estrogen's effects on the growth of breast cancer cells [44]. Cyclin D1, cyclin E, and cathepsin D expressions were upregulated in cells treated with triclosan, whereas p21 and Bax expressions were downregulated, and these gene expressions were blocked by kaempferol. This compound also inhibited 17β-estradiol and triclosan-induced expression of pIRS-1, pAkt, and pMEK1/2 [44]. Similar results to in vitro studies on tumor growth inhibition were observed in the in vivo xenografted mouse model [44]. In addition to ER-agonist, this phytoconstituent was known as AHR-antagonist (aryl hydrocarbon receptor), which was related to the inhibition activity on transcription of AHR in ER-α-negative breast cancer cell lines independent of ER-α expression [45]. Generally, the biphasic responses (via ER-dependent and ER-independent pathways) of kaempferol on.

Estrogen receptors were reported, which explained the role of this compound in regulating the body’s estrogenic activity and its valuable potency in inhibiting estrogen imbalance diseases [46]. As an ERK activation inducer, kaempferol may also activate MEK1 and ELK1 (a substrate of the ERK). Another study has demonstrated that they effectively reduce breast cancer cell viability [47]. The suggested chemoprotective mechanism of kaempferol can result in toxicity and proliferation arrest due to the downregulation of glucose transporter 1 (GLUT1) gene expression and inhibition of cellular glucose uptake in cancer cell lines. Furthermore, this flavonol structure could inhibit the cancer cell's monocarboxylate transporter (MCT1)-mediated lactate reuptake and destroy [48]. Downregulation of the matrix metalloproteinase-9 (MMP) expression and activity was the other process in breast cancer invasion treated with kaempferol using the MDA-MB-231 cell line [49]. This component inhibited AP-1, PMA-induced MMP-9 production, PKC-δ translocation, and MAPK signaling [49]. Furthermore, treating MCF-7 cells with kaempferol influenced the aryl hydrocarbon receptor in inhibiting CYP1A1 transcription, and CAT transcription was likewise suppressed by kaempferol. Moreover, kaempferol totally reduced the induction of 2,3,7,8-tetrachlorodibenzo-p-dioxin in band shift [50].

Cancer stem cell markers, such as Oct-4, Nanog, ABCB1, and ALDH1A1, were significantly reduced in MCF-7 cells treated with kaempferol and docetaxel. This study reported kaempferol as a more effective anti-cancer agent than docetaxel [51]. Kaempferol was a suppressor of primary tumor growth and lung cancer metastases in a mouse breast tumor model [52]. It reduced the expression of H3-cit (citrullinated histone H3) as a neutrophil extracellular traps biomarker (NETs), involved in NADPH/ROS-NETs signaling, and reduced tumor metastasis by inhibiting the ROS-PAD4 pathway [52]. Kaempferol could also suppress the proliferation of triple-negative breast cancer, contribute to the G2/M arrest induction, induce apoptosis and DNA damage, increase the expression of γ-H2AX and cleave the caspase-9, caspase-3, and p-ATM in comparison with the control group [53].

According to previous studies, various isolated kaempferol derivatives from the plant species, such as prenylated kaempferol and kaempferol-3*-O*-rhamnoside, also have noticeable inhibitory effects on breast cancer cells [54, 55]. Activating the caspase cascade signaling system could suppress breast cancer cells (MCF-7) growth and promote the apoptotic process in breast cancer cells [55]. Moreover, according to a recent study, a conjugate form of 1-deoxynojirimycin and kaempferol linked by an undecane chain revealed significant lipophilicity, anti-proliferative, and anti-α-glucosidase activities. The new derivative showed downregulation of the expression of COX-2, inhibited migration, decreased intracellular ROS and calcium cation levels, decreased Bcl-2 expression, and increased Bax expression in MCF-7 cells [56].

The other factors influencing the kaempferol mechanism of action in breast cancer are its pharmaceutical formulation and particle size, considered in two previous studies. The gold nanoparticles (AuNPs) and nanostructured lipid carriers (NLCs) of kaempferol are suggested as powerful drug delivery techniques in the management of breast cancer cells through the antioxidant, anti-proliferative, and antiangiogenic properties [57, 58].

### Cervical cancer

Cervical cancer was the fourth most often diagnosed and the fourth major cause of cancer mortality in women globally in 2020, with over 604,000 new cases and 342,000 fatalities [42]. Kaempferol impacted the glucose uptake and inhibited mitochondrial respiration in HeLa cells (derived from cervical cancer cells). In addition, HeLa cells activated autophagy as a survival response to this bioflavonoid. This study revealed that autophagy was related to early activation of the AMPK/mTOR-mediated pathway [59]. Apoptosis and cell cycle arrest was observed in HeLa cells when Kaempferol-7-O-b-D-glucoside, an extraction of *Smilax china* L. rhizome, was tested. This glycoside also reduced NF-*κ*B nuclear translocation, increased Bax protein, and decreased Bcl-2, indicating a mitochondrial pathway in apoptosis [60].

### Colon cancer

The GLOBOCAN 2020 research showed that rates of colon cancer are much higher in transitioned countries than in transitional countries. However, there is no significant difference in mortality rates because of the greater mortality in transitioning nations [42]. A previous study on the kaempferol effects on three colon cancer cell lines (MSU-2, HCT116, and KNC) with diverse expressed gap junction genes and function profiles concluded the possibility of anti-cancer properties with different functions [61]. The results of evaluations on KNC cells (it is expressed in connexin43 mRNA but lacked in gap junctional intercellular communication (GJIC) and Cx43 expression) indicated the ability of this flavonol structure on the induction effect of alkaline phosphatase (ALPase), reduction on Stat3, restoration of connexin43 protein (Cx43) phosphorylation, and functional GJIC. The Jak/Stat3 signaling pathway suppression was suggested as the important mechanism in the cells mentioned above. According to the results of this experiment among three different cell lines (MSU-2, HCT116, and KNC), kaempferol is considered a cytostatic component for only KNC cells [61]. According to Lee et al. studies, kaempferol made a series of molecular changes in the growth inhibition and HT-29 colon cancer cell line apoptotic function. In addition to a decrease in Bcl-xL and intact Bid, as well as an increase in Bik, Bad mitochondrial proteins, and the activation and cleavage of caspase-9, apoptosis function was caused by changes in the Bcl-2 family protein levels and localization (such as the reduction of Bcl-xL and intact Bid, as well as an increase in the Bik and mitochondrial Bad proteins).

Furthermore, the activation of caspase-8, the cleavage of caspase-3 and Bid, and the decrease of phospho-Akt and Akt activity by kaempferol led to Bad mitochondrial translocation [62]. Based on Budisan et al.'s studies, cell viability impairment, cell proliferation inhibition, apoptosis induction and autophagy, modifications in coding, and noncoding expression of genes have occurred in RKO and HCT-116 colon cancer Cell Lines treated with kaempferol [63]. Also, fewer cells in the G1 phase, but more in the G2 phase, were seen after kaempferol treatment [63].

The activation of death receptor 5 (DR5) by kaempferol increased the sensitivity of colon cancer cells to TRAIL-induced apoptosis in studies including medication combinations. In contrast, combining these two components didn't have significant cytotoxic effects on normal cells. Subsequently, the variety of kaempferol and TRAIL was introduced as a beneficial approach to cancer therapies [64]. Similar outcomes were observed in the kaempferol and 5-fluorouracil combination in HcT-8 and HcT-116 cell lines. The results indicated the synergistic effects of this bioflavonoid and 5-fluorouracil on cell viability, proliferation reduction, and apoptosis induction [65]. The molecular evaluation showed the upregulation of the Bax expression levels, downregulation of Bcl-2 and TS expression levels, and inhibition of the PI3K/Akt pathway [65]. A recent study reported that the combination of fluoxetine and kaempferol improved several biological properties such as the antioxidant, anti-inflammatory, and anti-proliferative effects with developed apoptotic activity and could exhibit a more potent chemopreventive effect against 2-dimethylhydrazine-induced preneoplastic lesion in rat colon than using fluoxetine alone [66]. Eventually, a recent experiment demonstrated that the combination of doxorubicin (DOX) and kaempferol was more efficient in induction of apoptosis and cytotoxic effect against HT-29 colon cancer cell line in comparison with each drug alone; also combined drug nanoformulations (polyethylene glycolated gold nanoparticles of doxorubicin-kaempferol) revealed a critical decreasing in the volume of colon tumor on the in vivo model without any severe side effects [67].

### Endometrial cancer

Endometrial cancer is the sixth most frequent malignancy in women and one of the most common malignancies in gynecology [68]. Nulliparity and metabolic abnormalities such as obesity have been linked to an increased incidence of this condition, which is one of the estrogen-dependent diseases [69]. Kaempferol inhibited the viability of Ishikawa and HEC 265 cells in previous research (estrogen receptor-positive, human endometrial adenocarcinoma cell line). At concentrations as low as 80 µg per milliliter, kaempferol inhibits the development of Ishikawa and HEC-265 cells. It induced the G2/M phase and suppressed the G1 phase. According to the results, this bioflavonoid structure led to apoptotic cell death in Ishikawa and HEC-265 cancer cells by decreasing endoplasmic reticulum (ER), survivin, and Bcl-2 levels and increasing p53 and PARP expression [69]. HEC108 cell line and growth of HEC180 were unaffected by kaempferol in the same research [69]. Using the human endometrial cancer cell line MFE-280 as a model, researchers found that inhibiting mTOR/PI3K/Akt signaling led to triggering apoptosis and halting cell cycle progression with substantial anti-cancer effects [70].

### Gastric cancer

There are around 1.1 million new cases of this disease each year, and 768,793 deaths from it are reported in the GLOBOCAN database, making it one of the most common cancers and the fourth-leading cause of death globally. Men have double the values of women [42]. There are several studies on the effects of kaempferol on different gastric cancer cells. In 2010, kaempferol revealed dosage and time-dependent inhibitory effects on MGC-803 cells, causing apoptosis and G2/M phase arrest [71]. Song et al. found that kaempferol promoted apoptosis and G2/M phase cell cycle arrest in gastric cancer cell lines, including MKN-28 and SGC-7901. Still, it had no impact on standard gastric epithelial cell lines, as previously reported (GSE-1) [72]. This investigation discovered that the xenograft tumor development was suppressed without any evident impact on the subjects' body weight, liver, or spleen. The G2/M cell cycle proteins, cyclin B1, Cdk1, and Cdc25C were reduced in kaempferol-treated cells, whereas Bcl-2 was reduced, and cleaved PARP, caspase-3, and -9 were enhanced. The expression of p-Akt, p-ERK, and COX-2 was reduced in MKN-28 and SGC-7901 cell lines [72]. The Korean researchers investigated kaempferol-mediated stomach cancer therapy's molecular mechanisms and biological activities in 2018. This bioflavonoid enhanced LC3-I to LC3-II transition, decreased p62 production, and activated autophagy and cell death through IRE1–JNK1-mediated Bcl-2–Beclin-1 interaction [73]. The outcomes also suggested HDAC/G9a pathway as another mechanism of kaempferol-mediated epigenetic changes for autophagy and cell death [73].

### Leukemia

Leukemia ranks 15th and 11th in terms of the prevalence of common world cancer and the leading cause of death worldwide, with approximately 500,000 and 300,000 cases reported for incidence and death rates, respectively, in 2020 [42]. Proliferation and maturation of hematopoietic myeloid cells are actively addressed in acute myeloid leukemia (AML) [74]. In addition to a poor prognosis and an undesirable treatment outcome, AML has several undesirable traits, such as higher recurrence rates and resistance to anti-cancer drugs [75, 76]. Kaempferol and quercetin might be used to more effectively inhibit the growth and viability of human leukemia THP-1 cells by targeting genes associated with survival [74]. Some studies say that kaempferol is hazardous for Jurkat T cells because it stops them from going through the G2/M phase and makes them die [77]. When kaempferol is administered to human HCT116 colon cancer cells, it stimulates the ataxia-telangiectasia mutated-p53 pathway, resulting in cell death [78]. However, when kaempferitrin enters HeLa cells, it causes apoptosis and has anti-cancer effects. Apoptosis was produced in promyelocytic leukemia cells by raising the Bax/Bcl-2 ratio and inhibiting MDR when kaempferol was administered [79]. Multi-drug resistance, apoptosis (PI3K and AKT), and differentiation (PML-RAR and HDAC1) were found to be upregulated in the study [79]. When the PI3K/Akt signaling pathways are inhibited, the growth of human leukemia cell lines (i.e., K562 and U937) is slowed, and their viability is reduced [74]. Tumor necrosis factor-related apoptosis-inducing ligand-resistant cells such as MOLT-4 have gained resistance to it via various mechanisms. Kaempferol enhanced the number of sub-G1 cells in the promyelocytic leukemia cells and triggered apoptosis. Also, it inhibited the production of FGF-8 and VEGFB, likely due to the reduction of survival-related signaling pathways [74]. According to real-time PCR, human leukemia K562 and U937 cell growth and viability are reduced when kaempferol inhibits PI3K/Akt signaling pathways [74, 79].

### Liver cancer

It is estimated that the world's most common primary cancer diagnosis and the third leading cause of cancer-related death will both be liver cancers in 2020 [42]. Additionally, there were more than 900,000 new cases and 800,000 deaths, with males having 2–3 times the prevalence and fatality rates [42]. A review of the previous research has shown that kaempferol affects liver cancer cells by affecting various molecular mechanisms and pathways in cancer progression. For example, Mylonis et al. revealed the influential role of kaempferol in inhibiting the viability of hepatoma (Huh-7) cancer cells, HIF-1, and MAPK in hypoxic conditions [80]. According to an in vivo study, kaempferol could modulate the levels of nucleic acids, membrane-bound ATPase (Na^+^/K^+^ ATPase, Mg^2+^ ATPase, and Ca^2+^ ATPase), mitochondrial TCA cycle, and carbohydrate metabolizing enzymes (hexokinase, phosphogluco-isomerase, aldolase, glucose-6-phosphatase, and fructose-1,6-diphosphatase) in aflatoxin B1 (AFB1) induced hepatocellular carcinoma. It was introduced as a potent anti-carcinogenic factor [81]. In another study, kaempferol acted as an anti-inflammatory agent. Its ability was proven to abolish the downregulatory action of TNF-α on liver-X-receptor alpha (LXR-α) in a dose-dependent manner [82].

Moreover, autophagy induction via upregulation of AMPK, downregulation of AKT and mTOR signaling pathways, and the induction of G2/M arrest through CDK1/cyclin B downregulation were observed in kaempferol-treated SK-HEP-1 cancer cells [83]. The assessment of different concentrations of kaempferol on ABCA1, mRNA expression levels in TNF-α stimulated HepG2 cell line demonstrated that although in the TNF-α-simulated inflammatory condition, kaempferol couldn't reduce the expression of ABCA1 rising back to untreated levels; high dose of this compound could lonely upregulated the ABCA1 mRNA expression considerably, which showed a positive relevance between ABCA1 and kaempferol. It was suggested that this bioflavonoid could be used as a suitable preventative agent in Tangier disease [84]. One of the main mechanisms proposed by Guo et al. in their in vitro investigation of HepG2 cells was the ER stress CHOP pathway, which increased the production of protein and mRNA of ER stress indicators [85]. The flavonoid structure was shown to promote apoptosis in hepatocytes in the rat model of hepatocellular carcinoma through the mitochondrial-dependent pathway by targeting upstream activities such as the enhancement of ROS formation, MMP downregulation, and the increasing of the caspase-3 activity in the cytosol [86]. Several pieces of literature described the protective effects of kaempferol on the liver against various oxidative stresses [87, 88]. By activating mitochondria and inhibiting the signaling pathways of PI3K/mTOR/MMP, kaempferol limits liver cancer cell proliferation and migration. And results showed that kaempferol didn't have any significant toxicity on normal cells [89]. This report also demonstrated a synergistic influence and more significant inhibitive effects of proliferation, migration, and invasion on liver cancer in combined treatment with doxorubicin and kaempferol. It was suggested that this bioflavonoid could interrupt cancer cell survival signaling pathways and may compensate for the shortcomings of monotherapies. Furthermore, the multi-targeting activity of kaempferol can reduce drug resistance during treatment [89].

Following Shakya et al.'s report, kaempferol protected the liver against alcohol- and thermally oxidized polyunsaturated fatty acid-induced oxidative stress [88]. Moreover, a recent study anticipated that kaempferol and kaempferide (kaempferol 4′-methyl ether) possessed significant effects on Nonalcoholic fatty liver disease (NAFLD) prevention and treatment, which might cause some necroinflammatory responses such as nonalcoholic steatohepatitis and consequently cancer [87, 90]. The induced molecular mechanisms of these two flavonol structures were reduced lipogenesis-related protein expression (e.g., SREBP1, FAS, and SCD-1). Moreover, they have decreased the expression of PPARγ, C/EBPβ, and two adipogenic transcription factors, HO-1 and Nrf2 [87]. Moreover, the molecular docking approach presented that the binding of kaempferol and kaempferid to SCD-1 could introduce an essential regulator in lipid metabolism [87]. Along with in vitro and in vivo experiments, in silico evaluations also confirmed the anti-liver cancer potential of kaempferol. The well-binding affinities to the receptor FKBP12-rapamycin binding domain and AKT serine/threonine-protein kinase concluded the improved efficacy in inhibiting mTORC1 compared with everolimus as a standard drug [91].

The studies above and several previous data show that kaempferol's different synthetic and natural derivatives also presented significant cancer-fighting effects against hepatocarcinoma cells [54, 92].

### Lung cancer

In 2020, greater and less than 2.0 million new cases and fatalities were due to lung cancer, respectively [42]. Cancer experts think that non-small cell lung cancer (NSCLC) is responsible for more than 11% of the worldwide cancer burden and more than 18% of all cancer fatalities [93]. The authors found that kaempferol significantly decreased in vivo lung metastasis using a realistic and uncomplicated B16F10 melanoma metastatic model. These inhibitory effects decreased MMP-9 synthesis and activity by reducing the MAPK and PKC signaling pathways. A549 cells die due to kaempferol's ability to inhibit the PI3K/AKT and ERK pathways. According to the results [94, 95], kaempferol suppressed A549 cells via activating the apoptosis pathway. A different test in NSCLC cells uses Nrf2 reporter luciferase as a biomarker (A549 and NCIH460). Nrf2 siRNA decreased NQO1 protein levels in A549 and NCIH460 cells [96]. In comparison to normal lung epithelial cells, NSCLC cells (A549 and NCIH460) express more significant amounts of Nrf2, as well as its downstream proteins NQO1 and HO1 (L132) [97]. At zero and 24 h following treatment with K-AuNCs, the researchers evaluated if kaempferol-conjugated AuNCs showed equivalent anti-migratory effects on A549 lung cancer cells. Kaempferol, toxic to lung cancer cells but not HK-2 human kidney normal cells, can be efficiently conjugated to AuNCs. It has been shown that K-AuNCs govern the development of A549 cells [98]. Pretreatment of A549 cells with kaempferol significantly decreased the toxicity of TGF-1. Through dephosphorylation of Smad3 at the Thr179 site in A549 lung cancer cells, kaempferol inhibits the EMT, migration, and MMP-2 activation induced by TGF-1 in these cancer cells. Kaempferol's ability to inhibit cancer cell development has been linked to estrogen-related receptors (ERRs) and its activity [99]. Finally, the fact that A549 lung cancer cells were resistant to TGF-1-induced EMT, migration, and activation of MMP-2 suggests that kaempferol's antimetastatic effect may target essential components of numerous signaling pathways implicated in tumor metastasis. In addition, in A549 cells, kaempferol-induced apoptosis seems to need MEK-MAPK activation, which acts before caspase-7. For the A549 lung cancer cell line, kaempferol is toxic to the cells. Kaempferol enhances the MEK/MAPK, affects the Bcl-2 proteins, and inhibits the phosphorylation of Akt1 to activate apoptosis [100]. By cleaving PARP and caspase-7, the MEK1/2 inhibitor prevents kaempferol-induced apoptosis. A549 cell death requires MEK-MAPK activation, which occurs before caspase activation, according to the literature findings [94].

### Nervous system cancer

According to the 2020 GLOBOCAN report, brain and nervous system cancer comprised more than 308,000 and 251,000 new cases and deaths, respectively [42]. It has been shown that the combination of kaempferol and TRAIL might be an essential strategy for treating glioma by suppressing survivin protein degradation [101]. Apoptosis was reduced when survivin was overexpressed in the cell. Kaempferol was also responsible for downregulating phosphorylated Akt and reducing the quantity of survivin protein to stop survivin [101]. It was discovered in this study that TRAIL and kaempferol were able to imitate caspase-8 cleavage while also having an anti-apoptotic effect that was not inhibited by survivin, which has been demonstrated in earlier research not to affect caspase-8 activation. As well as this, the apoptosis inhibitory Bcl-2, Bcl-xL, and Mcl-1 family members were all reduced in concentration by kaempferol [101]. Kaempferol was also shown to elicit caspase-dependent pathways, including the downregulation of XIAP and survivin, which ERK and Akt regulate in the other investigation on A172 human glioma cells [102]. Kaempferol was also shown to have neuroprotective properties against 4-hydroxynonenal-induced apoptosis in PC12 cells. This natural flavonoid prevented the 4-hydroxynonenal-induced apoptosis, linked to nuclear condensation, Bcl-2 downregulation, and proapoptotic caspase-3 activation. Additionally, it prevented phosphorylation of c-JNK in response to 4-hydroxynonenal, which was bound to p47phox, and inhibited NADPH oxidase activity in response to 4-hydroxynonenal (NOX). Results indicated that kaempferol might be a preventative agent against NADPH oxidase-mediated neurodegenerative disorders [103].

In 2018, an investigation on IMR32 and Neuro2A cells (human and mouse neuroblastoma cell lines), showed this flavonol structure induced neuroblastoma differentiation, decreased cell viability, promoted apoptosis, modulated the IRE1α (inositol-requiring enzyme one alpha) expression, and activated the IRE1α endoribonuclease [104].

### Ovarian cancer

As mentioned in the GLOBOCAN report, ovarian cancer has been considered a severe women malignancy, with 313,959 new cases in 2020 worldwide [42]. Based on the previous studies on OVCAR-3 (mutant p53), wild-type p53 of A2780/CP70 cells, anti-angiogenesis, and the significant inhibitory effects of kaempferol on VEGF (Vascular endothelial growth factor) gene expression via both Akt/HIF and ESRRA pathways were described [105]. The other study confirmed tumor anti-angiogenesis effects of kaempferol and added the induction effects of this flavonoid compound on G2/M cell cycle arrest through the Chk2/Cdc25C/Cdc2 and Chk2/p21/Cdc2 pathways in A2780/CP70 ovarian cancer cells [106]. The researchers described that kaempferol could increase the DR5 and Fas expression level and stimulate the extrinsic apoptosis pathway through the death receptors/FADD/Caspase-8 and Chk2 was not a responsible factor for the apoptosis induction and upregulation of p53 by kaempferol [106]. Kaempferol also displayed anti-proliferative properties on several human ovarian cancer cells (Caov-3, TOV-112D, SKOV-3, and OVACAR-3) by triggering autophagy and apoptosis G0/G1 cell cycle arrest and inhibition of MEK/ERK and STAT3 pathways [107]. In a recent study on A2780 cells, kaempferol effects increased cell apoptosis and decreased viability and proliferation. Furthermore, rising GRP78, PERK, ATF6, IRE-1, LC3II, beclin 1, and caspase-4 levels stimulated the cytotoxic endoplasmic reticulum/ autophagy mechanism, which was related to enhancing intracellular calcium cation levels. Cancer cells were made more sensitive to cisplatin by kaempferol, which did so via lowering the protein p-Akt in the cells [108]. Various studies have suggested using kaempferol combined with cisplatin to control the drug resistance problem in ovarian cancer [106, 108]. Earlier studies have also shown the synergistic effects of kaempferol and cisplatin inhibiting ABCC6 and cMyc gene transcription in ovarian cancer cells [109]. It should be noted that nano-formulations of kaempferol had a more influential role in reducing the survival of ovarian cancer cells than kaempferol alone [110].

### Pancreatic cancer

Affecting both men and women throughout the world, pancreatic cancer has a bleak outlook, making it one of the most dangerous and lethal tumors to develop [42]. Scientific research on three human pancreatic cancer cell lines (Miapaca-2, Panc-1, and SNU-213 cells) presented the anti-cancer effect of kaempferol via viability reduction and apoptosis increasing in a dose-depend manner [111]. This study introduced kaempferol as an anti-viable, antioxidant, anti-migration agent with no toxicity in low doses. It could mediate the anti-cancer activities against pancreatic cancer by blocking EGFR-related Src, ERK1/2, and AKT signaling pathways. Two glucoside derivatives of kaempferol (kaempferol-3-O-glucoside and kaempferol-4’-O-glucoside) didn't show any anti-cancer effects in the mentioned study [111].

Moreover, a recent study showed that kaempferol could successfully suppress pancreatic cancer in vivo and in vitro models. Akt/mTOR signaling activation might diminish the TGM2 mRNA and protein levels and ROS generation. Raising the expression of tissues transglutaminase is the cause of downregulation of ROS production, inhibition of related signaling pathways, and poor prognosis in pancreatic ductal adenocarcinoma [112].

### Prostate cancer

Prostate cancer is anticipated to be the second most common cancer in men and the fifth leading cause of cancer deaths globally by 2020, with an estimated 1.5 million new cases and 0.4 million fatalities [42]. As mentioned above, even though the inhibition of cancer cell growth and apoptosis induction were the main mechanisms of kaempferol, promoting the body's immunity also plays an essential role in fighting against various types of cancer. As an example, an investigation of the effects of kaempferol on the production of granulocyte–macrophage colony-stimulating factor (GM-CSF) in prostate cancer cells (PC-3) demonstrated the increasing of GM-CSF release by PC-3 kaempferol -treated cells without any disturbance on the mRNA levels could improve the activation of the antigen-presenting dendritic cells and the host immune system and consequently could develop the tumoricidal immune response. This natural flavonol stimulated the production of GM-CSF by activating the PLC, PKC, and MEK1/2 cascade [22]. The other literature study represented the apoptosis stimulating role of kaempferol in a dose-depend manner in the presence of dihydrotestosterone on LNCaP cells (androgen-sensitive human prostate adenocarcinoma cells). Moreover, it could inhibit the activities of dihydrotestosterone-induced androgen receptors and decrease the downstream targets of androgen receptors (e.g., PSA, TMPRSS2, and TMEPA1) and PSA protein levels, and also inhibit androgen receptor protein expression and nuclear accumulation. Eventually, kaempferol could suppress the vasculogenic mimicry formation and.

invasive potency in a dose-dependent manner [113]. Other research has shown that it may have an anti-proliferative impact depending on how much kaempferol-3-O rhamnoside is applied to LNCaP cells. For this reason, in LNCaP cells, it caused an increase in the expression of the enzymes caspase-8, -9, -3, and ADP-ribose polymerase[114].

### Skin cancer

Skin cancer, which develops when skin cells grow abnormally and unchecked, is one kind of cancer that may spread throughout the body. This malignancy is divided into two main groups keratinocyte cancers known as non-melanoma and melanoma [115]. According to the last GLOBOCAN data, over one million new cases with 63,731 death of non-melanoma (excluding basal cell carcinoma) and 324,635 new cases with 57,043 death of melanoma were reported in 2020 [42]. Based on the previous study of kaempferol-treated HaCaT cells, the results demonstrated 147 transcripts (including 18 upregulation and 129 downregulation) in cDNA microarray analysis that caused significant expression changes [116]. The results showed the role of kaempferol on the simulation of PPAR transcriptional activity in the HaCaT cell line, which transiently transfected with the PPRE-*tk*-Luc reporter gene, and suggested that its action mode could be mediated by PPAR pathways [116].

Moreover, recently published literature reported *Prunus cerasus* L. extract (sour cherry) as a source of essential polyphenols such as kaempferol, quercetin, and chlorogenic acid during apoptotic cell death of HaCaT cell line exposed to airborne particles with a diameter less than 10 µm. Cell viability decreased, reactive oxygen species were inhibited, and apoptosis-related gene expression (e.g., Bax, Bcl-2, and caspase-3) was inhibited due to the activation of transcription factor NF-κB [117]. The other experiment revealed that kaempferol significantly inhibited EGF-induced neoplastic transformation in JB6 P1 mouse epidermal cells. In addition, it could suppress AP-1 and NF-κB activation and PI3K/Akt signaling pathways, and the binding of kaempferol to PI3K inhibited UVB-induced activities [118]. Moreover, this natural compound targeted RSK2 and MSK1 to suppress and prevent UV radiation-induced skin cancer [119]. Kaempferol suppressed cell migration and apoptosis in the presence of melanoma malignancy, as well as downregulating the levels of mTOR, phosphorylated (p) mTOR, PI3K, and Akt proteins [120].

### Others

We summarized their information in this section because of fewer published experimental data for kaempferol effects on the other types of cancer cells. A study performed on the effects of kaempferol on head and neck squamous cell carcinomas revealed that this compound could inhibit the rate of oxygen consumption and reduce the content of intracellular ATP in tumor cells, which could induce anti-proliferative activities and tumor cells apoptosis and also could inhibit the capacity of migration. Furthermore, the results showed that combining this bioflavonoid and cisplatin could improve cisplatin resistance limitation in mentioned cancer cells [121].

On esophageal squamous cell carcinoma, kaempferol showed significant inhibitory effects on tumor cell proliferation and clone creation in an experiment for colony formation and cell colony formation. Kaempferol induced the G0/G1 phase arrest, changes in protein expression involved in cell cycle regulation, and inhibits tumor glycolysis in tumor cells. The epidermal growth factor receptor (EGFR) signaling pathway was reported as the possible mechanism for described function [122].

Apoptosis was activated, and cell cycle arrest was seen in 786-O and 769-P cells treated with kaempferol discovered by Song et al. They found that this bioflavonoid inhibited the EGFR/p38 MAPK signaling pathways, increased p21, decreased cyclin B1, stimulated PARP cleavages, induced apoptosis, and inhibited cell proliferation in human renal cell carcinoma (RCC) cells [123].

Kaempferol also reduced MMP-9 production by inhibiting NF-κB and AP-1 activation in the HT1080 (human fibrosarcoma) cell line, which was associated with a substantial decrease of IκBα and JNK phosphorylation that was driven by phorbol-12-myristate-13-acetate [124]. These results demonstrated the chemopreventive effects of kaempferol in controlling the inflammation risk, tumor invasion, and metastasis involving MMP-9 [124].

Also, in vitro and in vivo studies revealed that kaempferol suppressed cholangiocarcinoma development and metastasis [125]. According to the in vitro experiments, this natural polyphenol inhibited HCCC-9810 and QBC939 cells' proliferation and colony formation while inhibiting their migration and invasion capabilities. It was found that kaempferol decreased Bcl-2 expression and elevated Bax, Fas, cleaved-caspase-3, -8, -9, and PARP expressions. Furthermore, the phosphorylated AKT, TIMP2, and MMP2 were downregulated by kaempferol treatment [125]. The lung metastasis model saw fewer metastatic foci and lighter mice [125]. Moreover, decreasing the amount of Ki-67-positive cells was reported as a kaempferol effect [125].

According to previous research on retinoblastoma SO-RB50 cells, kaempferol affected the ERR-α. The Wnt/ β-catenin signaling pathway stopped cells from making more copies of themselves and spreading. This report demonstrated the occurrence of G2/M arrest and apoptosis in the SO-RB50 cell line [126].

The extrinsic mechanism of apoptosis was used to show that the kaempferol derivative known as 3-O-b-isorhamninoside, isolated from *Rhamnus alaternus* L. leaves, caused apoptosis in human lymphoblastoid cells. The obtained results represented that the induced apoptosis of the mentioned glycoside of kaempferol was related to the stimulation of caspase-3 and PARP cleavage [127].

A previous in vitro study indicated that kaempferol and its glycoside (kaempferol.

7-O-rhamnoside) inhibited PD-1/PD-L1 interaction, closely related to the T cell functions impairment and escape cancer cells from the immune surveillance [128]. In addition, decreasing the ERR-regulated target gene expression, such as reducing TFAM, Mfn2, Mn-SOD, and Cu/Zn-SOD gene expression and antagonizing the EER-α and -γ were related to the kaempferol effects on inhibiting mitochondrial biogenesis and cancer cells development [129].

### The combined action of kaempferol with other chemicals

Previous research suggests that coordinating flavonoids with transition metal ions may increase the anti-cancer activity of flavonoids. According to the results of an MTT test, kaempferol-Zn was in a better position than free kaempferol to inhibit the growth of cancer cells and reduce their viability. These findings provided sufficient evidence to support the hypothesis that kaempferol-Zn has the potential to trigger apoptosis in EC9706 cells. Consequently, the mitochondria were further damaged by kaempferol-Zn, which led to cell death [129]. Colon cancer cells were treated with a PEGylated AuNPs-DOX@Kaempferol drug delivery system. This system relied on chemically modified PEGylated AuNPs and electrostatic hydrogen-bonding interactions. As the pH became more acidic, the DOX and kaempferol molecules were freed from their critical interactions, and medications could also be released at intracellular locations. It resulted in a more significant antitumor effect and promoted death in colon cancer cells [130].

An investigation presented quercetin and kaempferol's cytotoxic action on HCT-116 cells; some green foods and fruits might contain equivalent amounts of both flavonoids. HCT-116 cells were prevented from entering the G2/M phase of the cell cycle by quercetin and kaempferol's chemopreventive effects, which resulted in a loss of viability in the cells when the two compounds were combined [131]. Chloroquine or sodium phenylbutyrate was added to kaempferol-treated ovarian cancer cells to reverse the effects of kaempferol on ovarian cancer cells (ER stress inhibitor). According to the results of this research, using kaempferol, either alone or in conjunction with cisplatin, may be an effective way to reduce ovarian cell tumorigenesis [132]. PANC-1 and BxPC-3 were inhibited most effectively when kaempferol and erlotinib were used together. An IHC staining confirmed the in vitro results that kaempferol and erlotinib further decreased the expressions of p-EGFR and AKT, as well as p-ERK1/2 and Bax when used together [133]. A critical factor in nanoparticle anti-cancer efficacy is particle size since nanoparticles enter cancer cell walls through increased permeability and retention (EPR). Compared to kaempferol on its own, treating cancer cells with nanoparticles loaded with kaempferol increased early apoptosis by between 0.1 and 6%. The cytotoxicity of paclitaxel was increased when combined with kaempferol-loaded NLCs against MDA-MB-468 murine breast cancer cells [134].

According to a recent study, NANOG, SOX2, MDR1, and CD44 are all induced pluripotent stem cell markers that may be reduced by kaempferol alone or in conjunction with Verapamil. Cancer cells’ survival and acquisition may be slowed by pharmaceutical combinations that interfere with the CD44–NANOG–MDR1 feedback loop activation network, according to a new study [135]. The kaempferol could be effectively conjugated to the AuNCs. The resultant conjugation was selectively harmful to the lung cancer cells while remaining non-toxic to the normal human kidney cells (i.e., HK-2 cell line). The clonogenic experiment provided conclusive evidence that K-AuNCs were responsible for controlling the growth of A549 cells. The findings offer credence to the natural flavonoid kaempferol's potential as an anti-cancer agent in conjunction with AuNCs [98]. The effects of kaempferol are amplified through the downregulation of cMyc, resulting in ovarian cancer cells being more likely to undergo apoptosis due to cisplatin treatment. It was shown that combining kaempferol and cisplatin might synergistically diminish ovarian cancer cells' vitality. The reduction in cell viability was achieved by blocking the gene transcription of ABCC6 and cMyc [136]. The research presents the findings of kaempferol-cisplatin-doxorubicin combo investigations. The results demonstrated a substantial synergistic therapeutic benefit when doxorubicin or cisplatin was used in conjunction with kaempferol in HCT-15 and MDA-MB-231 cell lines [137].

**Fig. 2:**
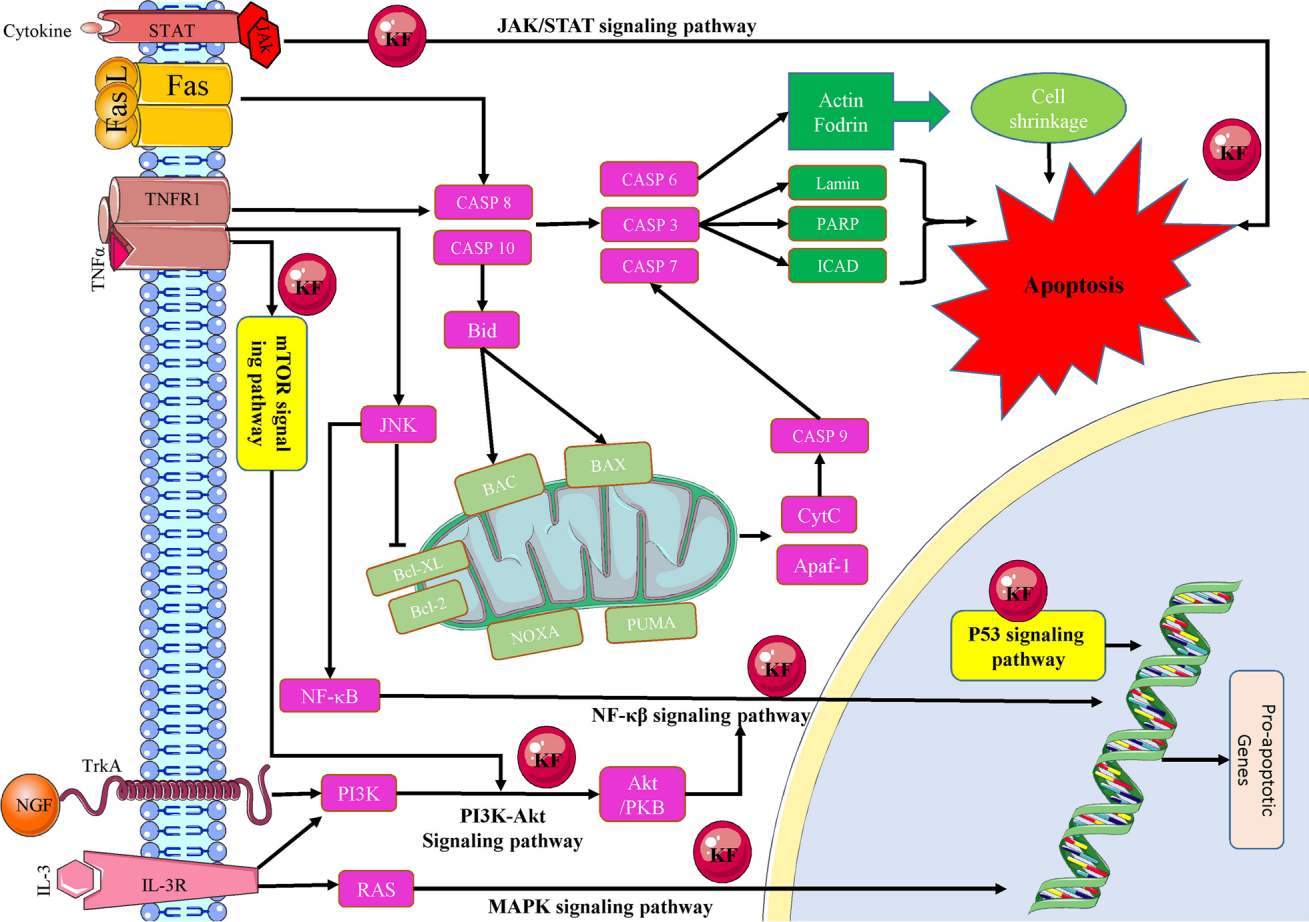
The activation tumour cell apoptosis using kaempferol as a therapeutics agent through diverse range of signaling pathways and mitochondrial mechanisms (KF: kaempferol)

As a result, the regulation of complex biological illnesses may be improved by using medication combinations that co-act with one another. In addition to controlling these potential pharmacological actions, kaempferol has also been found to induce other organ protective benefits, as listed in Table 1. Activation of apoptosis and mitochondrial-related pathways and processes has been shown in some studies to be the mechanism by which kaempferol kills cancer cells (Fig. 2).

**Table 1:** Characteristics of twenty one types of cancers in terms of treatment, study type, cell line, sample, dosage, treatment period, and mechanisms and possible involved signaling pathways

Cancer types	Treatment	Study	Cell Line	Sample	Dosage	Treatment Period	Mechanisms/Signaling Pathway	Ref
Bladder cancer	Kaempferol	In vitro	EJ	–	20–160 µM	24 and 48 h	Activation of Caspase-3	[35]
	Kaempferol	In vitro In vivo	5637 and T24	6 to 8-week old athymic BALB/cnu/nu male mice (subcutaneous xenografted mouse models)	In vitro: 50–150 µM In vivo: 50–150 mg/kg	48 h Intraperitoneal injection, daily, for four weeks	c-Met/p38 signaling pathway	[36]
	Kaempferol	In vitro In vivo	5637 and T24	5-week-old BALB/c nude mice (nude mice bearing tumor xenografts)	In vitro: 40 µM In vivo: 150 mg/kg	24 and 48 h Intraperitoneal injection, daily, 31 days	Ubiquitin–proteasome pathway	[37]
	Kaempferol	In vitro	EJ	–	20–80 µM	48 h	Activating p53 signal pathway	[34]
Bone cancer	Kaempferol	In vitro In vivo	U-2 OS, HOB, 143B cells	BALB/cnu/nu mice (8 weeks old)	In vitro: 25–200 µM In vivo: 25 or 50 mg/kg	24 h Oral administration, daily, 40 days	Endoplasmic reticulum stress pathway and mitochondrial signaling pathway	[39]
	Kaempferol	In vitro	U-2 OS	–	25–100 µM	48 h	Blocking MAPKs and AP-1 signaling pathways	[40]
Breast cancer	Kaempferol	In vitro	ER-positive (MCF-7, T47D, and ZR-75) and ER-negative (MDA231)	–	17.5–70 µM	24 and 48 h	Reduction in the expression of both IRS-1 and cyclin D1	[43]
	Kaempferol	In vitro	T47D, BT549 and MDA-MB-231	–	10 µM	24 h	Inhibition of AHR-dependent transcription	[45]
	Kaempferol	In vitro	MCF-7, T47D and MDA-MB-231 cells	–	20–100 µM	48 h	ERK signaling pathway	[47]
	Kaempferol	In vitro	MCF-7	–	10–100 µM	24 h	Inhibition of MCT1-mediated lactate reuptake	[48]
	Kaempferol	In vitro In vivo	MDA-MB-231	6 to 8 weeks old male C57BL/6 mice	In vitro: 10–40 µmol/L In vivo: 50, 100, and 200 mg/kg	24 h Intragastric administration, daily, 21 days	Inhibition of MAPK signaling pathway	[49]
	Kaempferol	In vitro In vivo	MCF-7	Female BALB/c nu/nu mice, 6-week old	In vitro: 50–100 µmol/L In vivo: 100 mg/kg	96 h Subcutaneous Injection 2 or 3 times a week for six weeks	ER and IGF-1R signaling pathway	[44]
	Kaempferol	In vitro	MCF-7	–	5–700 µM	48 h	Downregulation of oct4, nanog, abcb1 and aldh1a1	[51]
	Kaempferol	In vitro In vivo	4T1	7 to 8 weeks female BALB/C mice	In vitro: 25 µM /L In vivo 40, 80, and 160 mg/kg	24 h Oral gavage, daily, four weeks	Inhibition of ROS-PAD4 pathway	[52]
	Kaempferol	In vitro	BT474 and MDA-MB-231 cells	–	50 µM/L	72 h	Induction of DNA Damage, Promotion of apoptosis	[53]
	Kaempferol-loaded nlcs and paclitaxel	In vitro	MDA-MB-468 cells	–	1–8 nmol paclitaxel and 10–160 μmol/l kaempferol	24 and 48 h	PI3K/Akt pathway and apoptosis pathway	[134]
	Kaempferol-verapamil	In vitro Ex-vivo	BCSC and MDA-MB-231 cells	34 tumor samples of patients	104.8 µM kaempferol and 5 µM verapamil	48 h	Chemoresistance pathways	[135]
	Kaempferol-doxorubicin and cisplatin	In vitro	MDA-MB-231 cells	–	32, 18 and 9 µg/ml	24 h	signaling pathways such as apoptosis and angiogenesis	[136]
Cervical cancer	Kaempferol	In vitro	HeLa	–	100 and 200 µM	48 h	AMP-activated protein kinase-dependent autophagy	[59]
Cholangio carcinoma	Kaempferol	In vitro In vivo	HCCC9810 and QBC939	Male BALB/c athymic nude mice (4 weeks old)	In vitro: 30- 150 µM In vivo: 20 mg/kg	24, 48, 72 h Intraperitoneal injection, daily, Three weeks	Inhibition of PI3K/AKT pathway, apoptosis	[125]
Colon cancer	Kaempferol	In vitro	MSU2, HCT116, KNC	–	2.5, 5, 10, and 20 µM	24 h	Suppression of Jak/Stat3 signaling pathway	[61]
	Kaempferol	In vitro	HT29, SW480	–	20- 60 µM /L	24 and 48 h	Apoptosis and reduction of Akt activity	[62]
	Kaempferol	In vitro	RKO and HCT116	–	0.1–1000 µM	48 h	Upregulation of MMP28 and downregulation of NTRK3	[63]
	Kaempferol-TRAIL	In vitro	SW480	–	10–40 µM	24 h	Apoptosis	[64]
	Kaempferol- 5‑fluorouracil	In vitro	HcT8, HcT116	–	100 µM	24 h	PI3K/Akt signaling pathway	[138]
	Kaempferol-fluoxetine	In vivo	-	Adult male Sprague–Dawley rats	200 mg/kg	Oral administration, daily, 6 weeks	Decreasing ROS production, inhibition of lipid peroxidation	[66]
	Pegylated aunps-DOX@Kaempferol	In vitro	HT-29 cell line	–	50–100 nm	48 h	Intrinsic pathways	[130]
	Kaempferol-quercetin	In vitro	HT-116 cell line	–	1Q:1 K, 2Q:1 K, and 1Q:2 K	48 h	p53-caspase-3 pathway and pro-apoptotic Bcl-2 family members PUMA and Bax	[131]
	Kaempferol-doxorubicin and cisplatin	In vitro	HCT-15 cells	–	60, 30 and 15 µg/ml	24 h	signaling pathways such as apoptosis and angiogenesis	[136]
Endometrial	Kaempferol	In vitro	Ishikawa, HEC-265, HEC108, and HEC180 cells	–	36 µM, 72 µM	48 h	Suppression of the ER-α and the anti-apoptotic proteins	[69]
	Kaempferol	In vitro	MFE280	–	0.5–20 µM	24 h	Inhibition of mTOR/PI3K/Akt signaling pathway	[139]
Esophageal carcinoma	Kaempferol	In vitro In vivo	KYSE150 and Eca109 cells	Five-week-old female Balb/c athymic nude mice	In vitro: 30–60 μM In vivo: 100 mg/kg	24, 48, 72 h Intraperitoneal injection, every three days, 25 days	Inhibition of EGFR signaling pathway	[122]
	Kaempferol-zinc (II) complex	In vitro	EC9706 cells	–	6, 18, 30 µg/mL	24 h	Cellular signaling pathway	[129]
Fibrosarcoma	Kaempferol	In vitro	HT1080	–	10–100 μM	24 h	Blocking NF-κB	[124]
Gastric cancer	Kaempferol	In vitro In vivo	MKN28 and SGC7901	4-week-old athymic mice	In vitro: 25–200 μM In vivo: 20 mg/kg	24, 48, 72 h Intraperitoneal injection, daily, 3 weeks	Downregulation of G2/M cell cycle-associated proteins	[72]
	Kaempferol	In vitro	AGS, SNU216, NCI-N87, SNU638, and MKN74	–	25–100 μM	8, 16, and 24 h	Induction of autophagic cell	[73]
Head and neck	Kaempferol	In vitro	Cal27, HEp2, and DOK	–	70–280 μM	48 and 72 h	Induction apoptosis and inhibition of the capacity of migration	[121]
Leukemia	Kaempferol	In vitro	Human monocytic cell line THP1	–	40 µM	12, 24, 48, and 72 h	Significant decrease in Bcl-xL expression	[74]
	Kaempferol	In vitro	CCRF-CEM (CCL1199) and Jurkat E61 (TIB152)	–	10 µg/mL	4, 12, 24, 48, 72, and 96 h	Production and expression of IL-2 cytokine	[140]
	Kaempferol	In vitro	Human promyelocytic leukemia cell lines, HL60 and NB4	–	12.5–100 μM	24, 48, and 72 h	Decreased viability of leukemia cells	[79]
	Kaempferol	In vitro	Jurkat/Neo cells and Jurkat/Bcl-2 cells	–	25–75 μM	36 h	Bak and PUMA upregulation	[77]
	Kaempferol	In vitro	MOLT4 cell	–	95 μM	12, 24, and 48 h	Induced apoptosis	[141]
Liver cancer	Kaempferol	In vitro	Huh7	–	0–100 μM	24 and 48 h	Inhibition of HIF-1 and MAPK	[80]
	Kaempferol	In vivo	–	Male wistar albino rats	100 mg/kg	Oral administration, daily, 28 days	Mitochondrial TCA cycle	[81]
	Kaempferol	In vitro	HepG2	–	1–20 μM	24 h	Suppression of the downregulatory action of TNF-α	[82]
	Kaempferol	In vitro	SK-HEP-1	–	25–100 μM	24 h	Autophagy induction via AMPK, AKT, and mTOR signaling pathways	[83]
	Kaempferol	In vitro	HepG2	–	5–100 μM	3, 6, 12, and 24 h	Activation of ER stress-CHOP pathway	[85]
	Kaempferol	In vitro	Hepatocytes (obtained from the rat liver of hepatocellular carcinoma)	Male Sprague–Dawley rats	2.5–100 μM	24 and 48 h	Release of cytochrome c via ROS generation before cytotoxicity ensued	[86]
	Kaempferol	In vitro	Huh7, Huh1, HepG2, HepG2.2.15, SK-Hep-1, PLC/PRF/5, HLE, HLF, and Hep3B	–	40 μM	24, 48, and 72 h	Activating of mitochondrial signaling pathways and inhibition of the PI3K/mTOR/MMP signaling pathway	[89]
	Kaempferol	In vitro	HepG2	–	1–20 μM	24 h	Upregulated the ABCA1 mRNA expression	[84]
Lung cancer	Kaempferol	In vitro	A549 and NCIH460	–	1–50 μM	24, 48, and 72 h	Downregulation of a unique inhibitor of the NF-κB pathway	[96]
	Kaempferol	In vitro	A549	–	5–20 μM	24 and 48 h	ERK1/2 signaling pathway	[129]
	Kaempferol	In vitro	A549	–	17.5–70 μM 14–112 μM	24 and 48 h	PI3K/AKT and ERK pathways in cell apoptosis	[95, 94]
	Kaempferol	In vitro	A549	–	10- 50 μM	24 and 48 h	Activation of MEK-MAPK signaling pathway	[94, 100]
Nervous system cancer	Kaempferol	In vitro	U87 and U251 cells	–	50–200 μM	48 h	Apoptosis	[101]
	Kaempferol	In vitro	A172	–	50–200 μM	6, 12, 24, 36, 48 h	Induction of cell death	[102]
	Kaempferol	In vitro	PC12	–	5 and 10 μM	24 h	Inhibiting activation of the NOX-JNK signaling pathway	[103]
	Kaempferol	In vitro	IMR32 and Neuro2A	–	50 μM	48, 72, 96 h	Induction of IRE1a -XBP1 pathway	[104]
Ovarian cancer	Kaempferol	In vitro In vivo	OVCAR3 and A2780/CP70 cells	Chick embryo chorioallantoic membrane (CAM)	In vitro: 10–80 μM In vivo: 20-*µ*M	24 h Single-dose, five days incubation	Downregulation of HIF-1α; repression of AKT phosphorylation	[105]
	Kaempferol	In vitro	A2780/CP70	–	30–50 μM	48 h	Caspase-8 pathway	[106]
	Kaempferol	In vitro	Caov3, TOV-112D, SKOV3, OVACAR3	–	12.5–50 μM	24 h	Upregulation of apoptotic proteins and STAT3 signaling pathways	[107]
	Kaempferol	In vitro	A2780	–	40 µmol/l	24 h	Activation autophagy mechanism	[108]
	Kaempferol-cisplatin	In vitro	A2780	–	40 µmol/l kaempferol with cisplatin (0, 2, 4, 6, 8, 10, 15, 20 µmol/l)	24 h	PI3K/Akt signalling pathway	[132]
	Kaempferol-cisplatin	In vitro	OVCAR-3	–	0–80 μM Cisplatin with other chemicals of 0–20 μM	24 h	Apoptosis caused by down regulation of cMyc	[98]
Pancreatic cancer	Kaempferol	In vitro	Miapaca2, Panc1, and SNU213 cells	–	0.005–200 μM	72 h	Inhibition of EGFR and AKT pathways	[111]
	Kaempferol	In vitro In vivo	PANC1, Miapaca2 cells	Tumor Xenograft models (male BALB/c mice)	In vitro: 2.5–1000 μmol/L In vivo: 25–100 mg/kg	48 h Gavage for seven days	Induction of ROS-dependent apoptosis via Akt/mTOR signaling	[112]
	Kaempferol- erlotinib	In vitro In vivo	PANC-1 and BxPC-3 cell lines	In vivo: Four-week-old female mice	Various dosages	In vitro: 72 h In vivo: 4 weeks	PI3K/AKT signaling pathway and epidermal growth factor receptor	[133]
Prostate cancer	Kaempferol	In vitro	PC3	–	10 μM	18 h	Activation of PLC, PKC, and MEK1/2 cascade	[22]
	Kaempferol	In vito	LNCaP, PC3	–	5- 15 μM	24, 48, 96, and 144 h	Inhibition of cell proliferation via androgen-dependent pathway	[113]
Renal cancer	Kaempferol	In vitro	786O and 769P	–	In vitro: 50- 150 μM	24, 48, 72 h	Inhibition of MAPK signaling pathways,	[123]
Retinoblastoma	Kaempferol	In vitro In vivo	SO-RB50	25 human tissue samples (included five normal, 11 normal pediatric retinas, and 14 retinoblastomas)	10–30 μM	24 h	Suppression of Wnt/β-catenin signaling pathway	[126]
Skin cancer	Kaempferol	In vitro	HaCaT	–	1–10 μM	24 h	PPAR pathways	[116]
	Kaempferol	In vitro	JB6 P + mouse epidermal cell line	–	10–40 μM	24 h	Inhibition of PI3K activity	[118]
	Kaempferol	In vitro In vivo	A431, A431 sh-RSK2, A431 sh-MSK1, or A431 sh-RSK2/ sh-MSK1, NIH3T3	Female SKH-1 hairless mice	In vitro: 0–50 μM/L In vivo: 0.5 or 1 mg	2 h Topical application, 12 weeks	Suppresses RSK2 and MSK1 kinase activities	[119]
	Kaempferol	In vitro	HaCaT	–	200 µg/mL of Prunus cerasus	24 h	Blocking of the mitochondrial pathway of apoptosis	[117]
	Kaempferol	In vitro	A375	–	10–80 μM	24, 48, and 72 h	Induction of apoptosis and downregulation of mTOR/PI3K/AKT pathway	[120]

The treatments include kaempferol as a single agent or being combined with erlotinib, cisplatin, zinc (II), doxorubicin, TRAIL, 5‑fluorouracil, fluoxetine, PEGylated AuNPs-DOX, quercetin, paclitaxel, verapamil

## Conclusion

Kaempferol is a bioflavonoid compound involved in reducing the incidence of hormone-related cancers. Oncogene-induced apoptosis and growth inhibition are the primary mechanisms of this compound; however, increasing the host's immunological response is also considered an important mechanism. When present in more significant quantities, kaempferol is associated with an anti-cancer action; yet, when present in lower concentrations, it is associated with a pro-cancer activity. Kaempferol may have a role in activating apoptosis in many different kinds of cancer cells. If this is the case, kaempferol may have an anti-cancer effect via intrinsic and extrinsic apoptotic pathways. Because of its propensity to limit cell proliferation, kaempferol is engaged in different processes. These activities include activation of JAK/STAT3, PI3K/AKT, and NF-κB, as well as interference with TNF-induced activation of MAPK. Considering the interaction induced by various elements such as gender and immunological conditions is necessary. In vitro and animal studies have been done to determine precisely what function kaempferol plays, but more research is needed to figure out the mechanism(s) by which it works in humans.

In summary, based on the literature that has been published so far, the use of kaempferol in combination therapy is successful. However, there is still a lack of sufficient scientific data and regulatory approval from the appropriate authorities. Kaempferol has been shown to have chemotherapeutic potential; however, further data and studies are needed to confirm kaempferol's status as a potentially effective chemotherapeutic drug. Preclinical evidence from human trials must also be obtained before using kaempferol as a chemotherapy drug. And if additional research is done on kaempferol's tumor growth-inhibiting capacity, it might be a valuable therapeutic drug in the future.

## Data Availability

Not applicable.

## Abbreviations

ABCA1: ATP-binding cassette transporter
ABCC6: ATP-binding cassette subfamily C member 6
AFB1: Aflatoxin B1
AIF: Apoptosis-inducing factor
AKT: Ak strain transforming
ALPase: Alkaline phosphatase
AhR: Aryl hydrocarbon receptor
AML: Myeloid leukemia
AMPK: Adenosine monophosphate-activated protein kinase
ATF6: Activating transcription factor 6
ATM: Ataxia telangiectasia mutated
AP-1: Activator protein 1
ATP: Adenosine tri-phosphate
BAX: Bcl-2-associated X protein
Bcl2: B-cell lymphoma 2
GM-CSF: Granulocyte–macrophage colony-stimulating factor
H2AX: H2A histone family member X
HDAC: Histone deacetylase
HIF: Hypoxia-Inducible Factor
HO-1: Heme oxygenase-1
IκBα: Nuclear factor of *kappa* light polypeptide gene enhancer in *B*-cells inhibitor, *alpha*
IRE: Inositol-requiring kinase
IRS: Insulin receptor substrate
JAK: Janus kinase
JNK: C-Jun N-terminal kinase
K-AuNCs: Kaempferol-conjugated gold nanoparticles
LC3: Microtubule-associated protein 1A/1B-light chain 3
PERK: Protein kinase RNA- like endoplasmic reticulum kinase
PCR: Polymerase chain reaction
PLC: Phospholipase C
PPAR: Peroxisome proliferator-activated receptor
PTEN: Phosphatase and tensin homolog
PI3K/Akt: Phosphatidylinositol 3-kinase/protein kinase B
PML/RARα: Promyelocytic leukemia/retinoic acid receptor-α
TNF: Tumor necrosis factor
Bcl-xL: B-cell lymphoma-extra large
Bid: A BH3 domain-only death agonist protein
BMI: Body Mass Index
BRCA: Breast cancer type
CAT: Chloramphenicol acetyltransferase
C/EBPβ: CCAAT/enhancer-binding protein β
Cx43: Connexin43
Caspase: Cysteine-aspartic proteases, cysteine aspartases
CHOP: CCAAT-enhancer-binding protein homologous protein
c-Met: Mesenchymal-epithelial transition factor
CYP1A1: Cytochrome P450, family 1, subfamily A, polypeptide 1
CDK: Cyclin-dependent kinase
Cdc25C: A cyclin of the specific phosphatase family that activates the cyclin B1/CDK1 complex
Chk2: The CHEK2 gene encodes for checkpoint kinase 2
COX: Cyclooxygenase
MAPK: Mitogen-activated protein kinase
MCF-7: Michigan Cancer Foundation 7
MDR: Multiple drug resistance
MEK: MAPK/ERK kinase
Mfn2: Mitofusin-2
Mcl-1: Myeloid cell leukemia 1
MMP: Matrix metalloproteinase
MSK: Mitogen and stress-activated kinase
MTT: 3-(4,5-Dimethylthiazol-2-yl)-2,5-diphenyl-2H-tetrazolium bromide
mTOR: Mammalian target of rapamycin
NADPH: Reduced nicotinamide adenine dinucleotide phosphate
NAFLD: Nonalcoholic fatty liver disease
PSA: Prostate-specific antigen
RSK: Ribosomal S6 kinase
ROS: Reactive oxygen species
SCD-1: Stearoyl-CoA Desaturase-*1*
SOD: Superoxide dismutase
SREBP1: Sterol Regulatory Element-binding Protein-1
Stat3: Signal transducer and activator of transcription 3
TUNEL: Terminal deoxynucleotidyl transferase dUTP nick end labeling
dDMPs: Deoxydinucleoside Monophosphates
DNMT3B: DNA methyltransferase 3B
EGFR: Epidermal growth factor receptor
ELK1: ETS Transcription factor ELK1
EMT: Epithelial-mesenchymal transition
ERK: Extracellular signal-regulated kinase
ER-negative: Estrogen receptor-negative
ERR-α: Estrogen-related receptor α
ESRRA: Estrogen-related receptor alpha
FADD: FAS-associated death domain protein
FKBP12: 12-KDa FK506-binding protein
FGF-8: Fibroblast growth factor 8
GRP78: Glucose Regulated Protein 78,000
GJIC: Gap junction intercellular communication
GLOBOCAN: Global Cancer Incidence, Mortality, and Prevalence
NF-*κ*B: Nuclear factor-kappaB
NOX: ADPH oxidase
NQO1: NAD(P)H quinone oxidoreductase 1
Nrf2: Nuclear factor erythroid 2-related factor 2
PAD4: Protein arginine deiminase 4
PD-1: Programmed cell death protein 1
PD-L1: Programmed death-ligand 1
pAkt: Phosphorylated Protein kinase B
Phox: Phagocyte oxidase
PMA: Phorbol myristate acetate
PKC: Protein kinase C
PARP: Poly (ADP-ribose) polymerase
TCA: Tricarboxylic acid cycle
TFAM: Mitochondrial transcription factor A
TGF: Transforming growth factor
TGM2: The transglutaminase 2 gene
TIMP2: Tissue inhibitor of metalloproteinases 2
TMEPA1: Transmembrane prostate androgen protein
TMPRSS2: Transmembrane Serine Protease 2
XIAP: X-linked inhibitor of apoptosis protein
RCC: Renal cell carcinoma
